# Impact of SARS-CoV-2 Genetic Blueprints on the Oral Manifestation of COVID-19: A Case Report

**DOI:** 10.1055/s-0041-1735538

**Published:** 2021-09-16

**Authors:** Amir Khodavirdipour, Mahsa Asadimanesh, Seyed Alireza Masoumi

**Affiliations:** 1 Department of Molecular Genetics, Faculty of Natural Sciences, University of Tabriz, Tabriz, Iran; 2 Private Practice, Hamedan, Iran

**Keywords:** COVID-19, SARS-CoV-2, oral manifestation

## Abstract

Nonsegmented positive-sense RNA enveloped RNA severe acute respiratory syndrome coronavirus 2 (SARS-CoV-2) virus can result in coronavirus disease 2019 (COVID-19). This virus is from β-coronaviridae family of viruses. The common signs and symptoms of COVID-19 include pyrexia, cough, dyspnea, fatigue, myalgia, cephalgia, diarrhea, and nausea. Physicians and dentists around the world could directly link the COVID-19 and oral diseases such as ageusia and anosmia. After time passes, different aspects of symptoms of the diseases have been discovered. Research suggests that the oral cavity is the most vulnerable region for the virus because of angiotensin-converting enzyme-2 (ACE2) receptor abundance in the mouth. In this case report (no. of patients = 6), we would like to report significant findings in patients who were diagnosed with COVID-19 reported to our clinic during May 2021 complaining about the oral manifestation of it such as xerostomia, gingival inflammation, and cracked teeth. All patients are younger than 40 years with no history of dental complaints and oral diseases. Fortunately, these symptoms are not life threatening and treatable/manageable by current treatment options. To date, there is no clear proof of how and via which pathway, SARS-CoV-2 genomic blueprint causes the oral manifestation of COVID-19 beside ACE2 receptor which is the only known biopathway for such incidents.

## Introduction

Coronavirus disease 2019 (COVID-19) shocked the world by reporting the first case after hiding the outbreak for months by late 2019, and it is called the first pandemic disease of the 21 century by World Health Organization and till date, the mortality rate on June 21, 2021, crossed 3.9 million and positive cases crossed 180 million since and growing day by day in countries such as Russia, Iran, Brazil, Mexico, and India with fragile health care systems and latest variations surfaced worldwide due to the mutations. 1 COVID-19's main mode of transmission is human to human by air droplets either via coughing, sneezing, or even speaking. To date, the best way of protection against the disease based on hygiene protocols is frequent hand washing, wearing a face mask, and keeping the safe social distancing from the people in public places. 2 The disease can be diagnosed by polymerase chain reaction (PCR) test and for its progress and lung invasion, computed tomography scan and chest X-ray shall be advised. The common treatment regimen is azithromycin 500, zinc, vitamin C, ciprofloxacin 500, and omeprazole. 3

## Result

### Patients, Methods, and Characteristics

Six individuals, four females and two males, all younger than 40 years, were included in the case report. All of them had PCR +ve test results for COVID-19 with mild lung involvement and back to normal life after 15 to 25 days. Around 5 to 8 days after the first sign of the infection, they have referred to us with oral complaints. Besides our symptomatic management treatment regimen, they have followed the infectious disease specialist regarding the treatment options.

## Discussion

Oral manifestation of COVID-19 shall be called one of the latest so-called COVID-19 symptoms. Current study proofing that besides known symptoms of COVID-19 including pyrexia, cough, dyspnea, fatigue, myalgia, cephalgia, diarrhea, nausea, anosmia, and ageusia, signs such as xerostomia, gingival inflammation, cracked teeth, oral ulceration and gingival breakdown, and anosmia and ageusia should be considered as COVID-19 diagnostic elements.

### Two Patients Reported with Gingival Inflammation

Inflammation and bleeding in tissues of the oral cavity have been suggestive of generalized elevation of inflammation because of the increase in interleukins (ILs) and cytokines started by the severe acute respiratory syndrome coronavirus 2 (SARS-CoV-2) virus. The severity of COVID-19 has been associated with dysregulation of the immune system, which helps in cytokines storm. Periodontal diseases shall increase circulating cytokines level, especially IL-6, which has been suggestive as one of the main cytokine storm elements. 4 Periodontal diseases are now being evaluated as a potential condition toward the severity of COVID-19.

### One Patient with Dry Mouth (Xerostomia)

COVID-19 has been a candidate to result in xerostomia for different reasons. The most routine cause is breathing by mouth because of wearing a face mask. Breathing by mouth shall dehydrate the oral tissues particularly in case of infrequent hydration. Research showing that other biopathways are involved in viral entry to the salivary glands, which are well known due to the abundance of angiotensin-converting enzyme-2 (ACE2) receptor. 5 More research is needed to determine the reason behind it. Meanwhile, dentists must note that dry mouth has been associated with an elevation in both *Candida* infection and caries.

### Two Patients with Gingival Tissue Breakdown and Oral Ulceration

COVID-19 has been linked to vascular anomalies because of viral damage to the blood vessels. This process can be described as a mechanism in which the virus acquires entry to the endothelial cells by ACE2 receptor and harm them, consequently leads them into hypoxia. Tissue necrosis such as oral ulceration shall be the consequence of damages to the blood vessel. Tissue damage and ulceration shall be additionally aggravating by elevated upregulation in inflammatory markers and inflammation itself because of SARS-CoV-2 virus. 6 Currently, case reports in the literature show that confirmed positive COVID-19 patients having oral ulceration are suggestive of being caused by SARS-CoV-2 virus. 7

### One Patient with Cracked Teeth

In September 2020, *New York Times* published a paper that showed the dentists observing an immense elevation in patients visiting them with fractured or cracked teeth since pandemic announcement. 8 The paper cited a huge increase in teeth grinding and clenching (bruxism) as the most likely reason. The above article explicitly examined three factors associated with the current pandemic which may affect the increases in bruxism. Psychological stress is named as the primary factor of stress-related cracked teeth. The next factor is wrong orthopaedic posture due to work from home during the COVID-19 pandemic which shall result in bruxism. And the last factor is obstructive sleep apnea and/or sleep deprivation would lead to cracked teeth and bruxism.

### Five Patients with Anosmia and Ageusia

Five out of six above patients reported the loss of smell and taste. The unanticipated onset of anosmia and ageusia are two signs that shall be the COVID-19 earliest indicator. Up to 80% of patients who were confirmed positive for the COVID-19 shall have personalized complaints of anosmia and ageusia, especially in mild or asymptomatic cases. 9 The reason behind the ageusia and anosmia is expected to be due to disruption of cranial nerves I, VII, IX, and X, and neural transmission supporting cells by SARS-CoV-2 virus. 10 Furthermore, due to the presence of innumerable ACE2 receptor on the tongue, direct entry into the tongue cells by the virus is possible.

## Strengths and Limitations

The results from this case report are limited by the fact that we were only able to obtain data from six patients. A larger group of patients would have been preferable. However, it is difficult due to COVID-19 pandemic situation.

## Conclusion

Age of the individuals, amount of virus inhaled and severity of disease are the key factors that determine the gravity of the COVID-19. Opportunistic infection, lack of oral hygiene, comorbid conditions such as kidney failure and diabetes mellitus, stress, hyperinflammatory responses, and vascular anomalies are the main elements that determine the severity of oral manifestation in COVID-19 patients. The main genetic pathway for such manifestation is the abundance of ACE2 receptors and the feasibility of the virus to insert its genetic material into the host's tongue cells.
